# Supplementary material for: An empirical examination of sample size effects on population demographic estimates in birds using single nucleotide polymorphism (SNP) data
Source: PeerJ. 2020 Sep 16;8:e9939. doi: 10.7717/peerj.9939 (PMC7501783; doi:10.7717/peerj.9939)
Supplement: Supplemental Information 1 [file peerj-08-9939-s001.docx]

## Supplementary Information

Table S1: Linear regression equations for scaled root mean square error (SRMSE) for each parameter in which breaking lineages into low-divergence and high-divergence groups improved the regression model, summarizing how accuracy declines with diminished sample sizes. Parameters are effective population size (*ν_1_* and *ν_2_*), time since divergence (*T*), and *Θ* (defined as 4*N_ref_μ*, where *N_ref_* is ancestral population size and *μ* is mutation rate per generation). Note that these are based on SMRSE values (to enable among-lineage comparisons; Table 3). Thus, *y* in the regression equation *y = mx + b* is SMRSE for that particular demographic variable (*m* is slope, *x* is *N*, and *b* is the *y* intercept).

| **SMRSE for variable** | **Regression equation (y = mx + b)** | **r^2^** | ***P*** |
| --- | --- | --- | --- |
| *ν_1_* (low-div group) | y = -0.04467 * *N* + 0.40622 | 0.304 | 0.0052 |
| *ν_1_* (high-div group) | y = -0.30251 * *N* + 1.68585 | 0.189 | 0.0299 |
| *ν_2_* (low-div group) | y = -0.10533 * *N* + 0.69169 | 0.355 | 0.0021 |
| *ν_2_* (high-div group) | y = -0.21646 * *N* + 1.25166 | 0.352 | 0.0018 |
| *T* (low-div group) | y = -0.04739 * *N* + 0.40888 | 0.395 | 0.0010 |
| *T* (high-div group) | y = -0.15276 * *N* + 0.99239 | 0.328 | 0.0028 |
| *Θ* (low-div group) | y = -0.02843 * *N* + 0.26395 | 0.202 | 0.0275 |
| *Θ* (high-div group) | y = -0.04878 * *N* + 0.43241 | 0.303 | 0.0043 |

Table S2. Genomes used for calculation of substitution rates and time since most recent common ancestor (TMRCA, from Claramunt & Cracraft, 2015), substitution rate, and generation time for each lineage in which demographic parameters are translated into biologically meaningful values in this paper.

|  | Genome | GenBank accession number | TMRCA (my) | Substitution rate (subs/site/gen) | Generation time (yrs) | Sources |
| --- | --- | --- | --- | --- | --- | --- |
|  |  |  |  |  |  |  |
| ***Tringa brevipes /T. incana*** | *Charadrius vociferus* | GCA_000708025.2 | 53.5153 | 2.453 x 10^-9^ | 4.88 | [(Gill, McCaffery, & Tomkovich, 2002)](https://paperpile.com/c/lPzM9a/2zsk) |
|  |  |  |  |  |  |  |
| ***Pinicola enucleator* subspp.** | *Zonotrichia albicollis* | GCA_000385455.1 | 21.7098 | 1.577 x 10^-9^ | 2 | [(Adkisson, 1999)](https://paperpile.com/c/lPzM9a/d0Fd) |
| ***Pica pica/Pica hudsonia*** | *Taeniopygia guttata* | GCA_000151805.2 | 41.5132 | 1.304 x 10^-9^ | 2.5 | [(Trost, 1999)](https://paperpile.com/c/lPzM9a/McqJ) |

## Supplementary Information figures


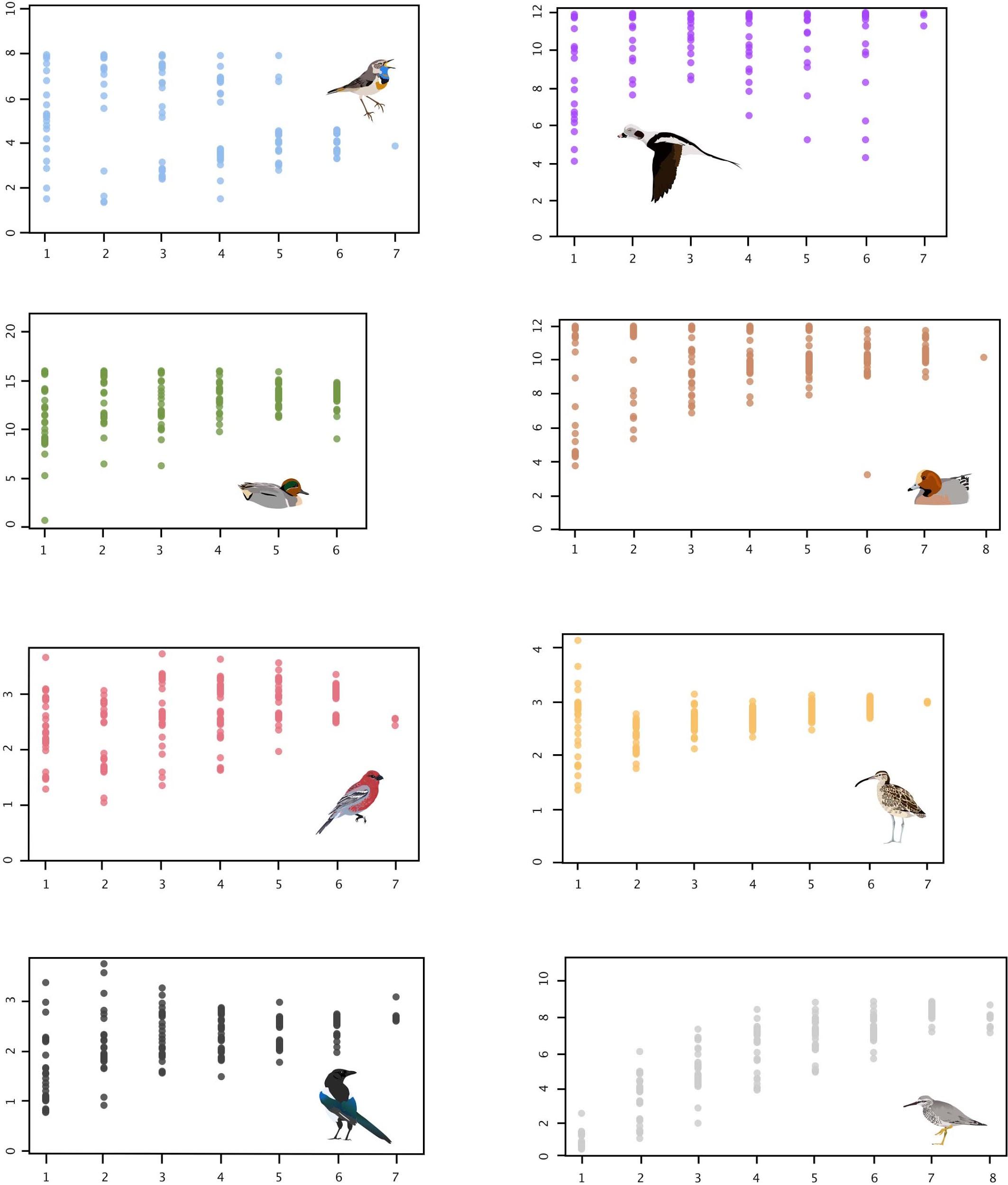


Figure S1: Estimates of *ν_1_* (effective size of population 1) at varying sample sizes in eight lineages (vertical axis is *ν_1_*, and horizontal axis is sample size as number of individuals). Parameters are raw, unconverted values directly from δaδi analyses.


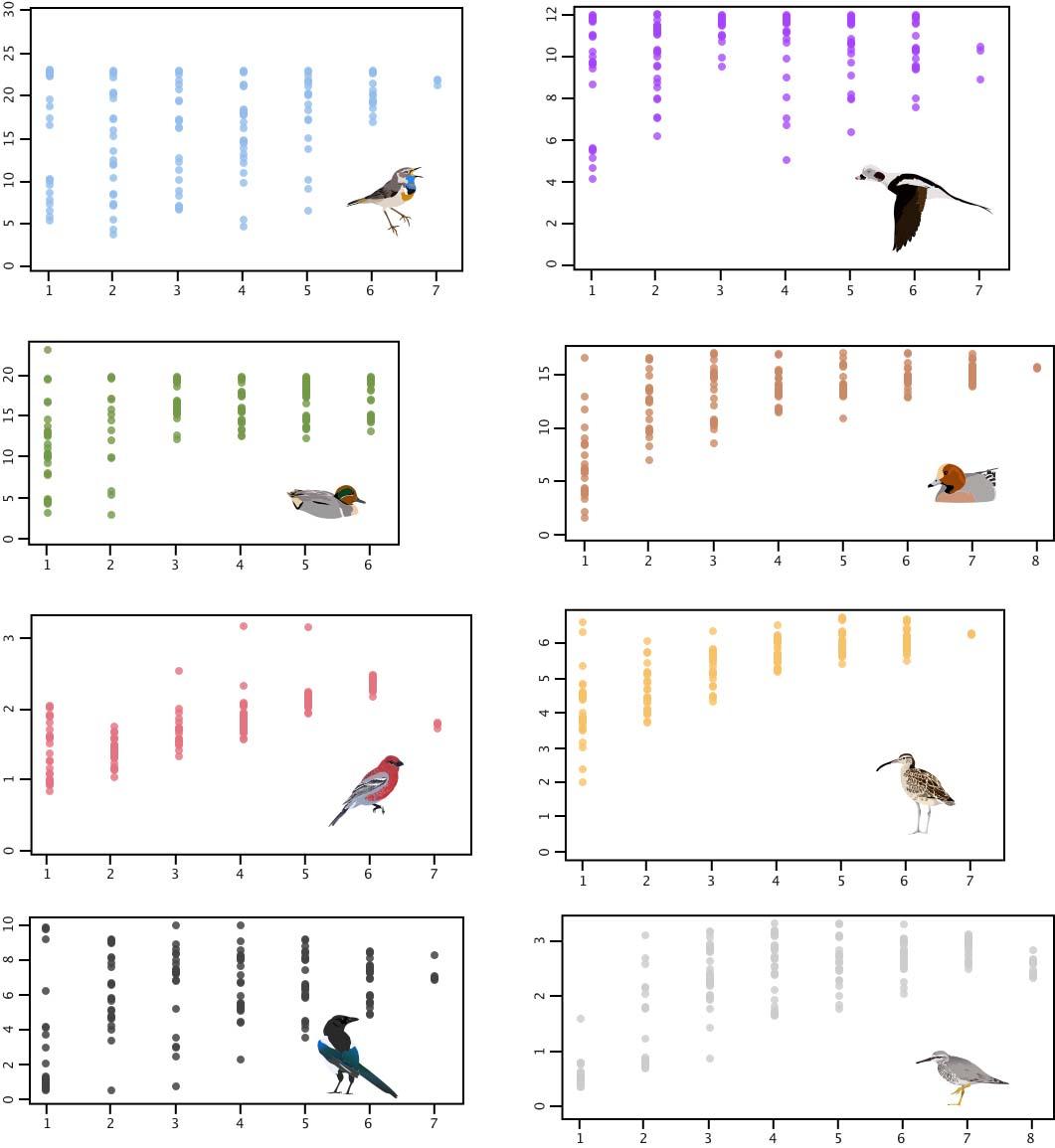


Figure S2**:** Estimates of *ν_2_* (effective size of population 2) at varying sample sizes in eight lineages (vertical axis is *ν_2_*, and horizontal axis is sample size as number of individuals). Parameters are raw, unconverted values directly from δaδi analyses.


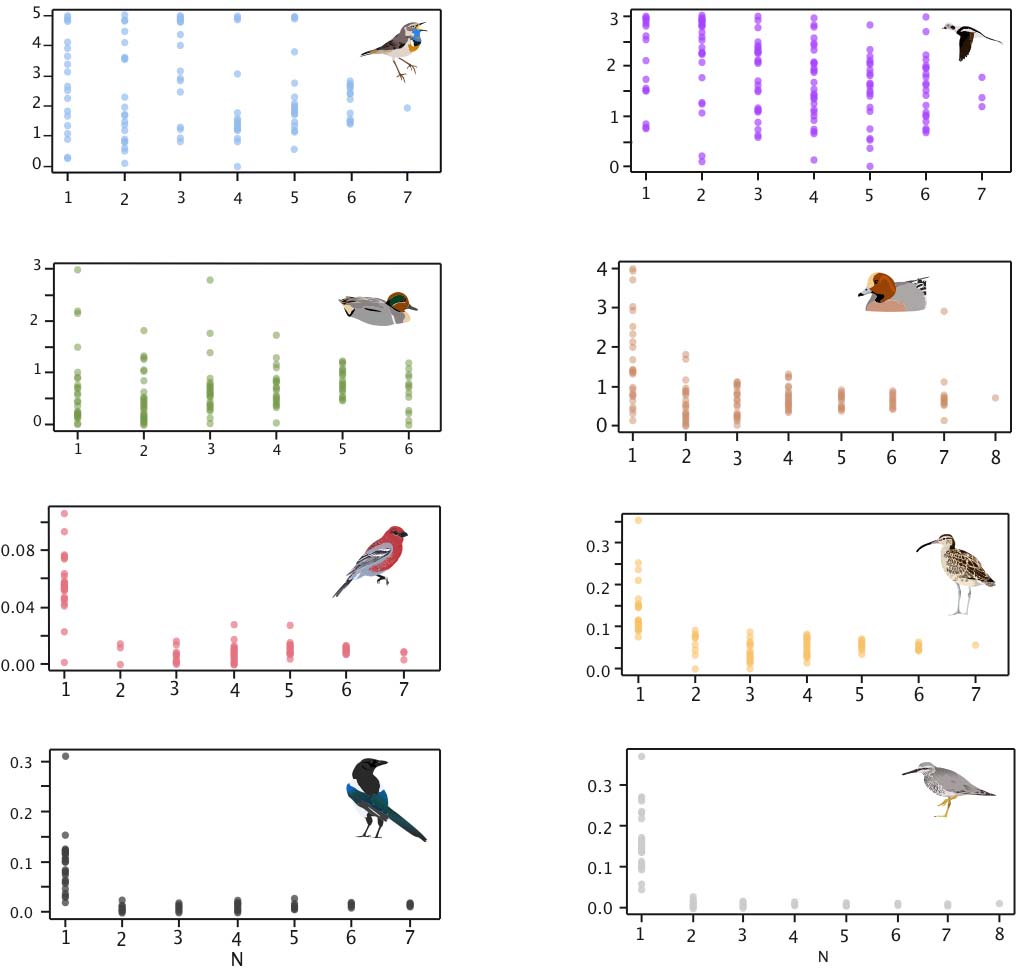


Figure S3: Estimates of *m* (migration or gene flow) at varying sample sizes in eight lineages (vertical axis is *m*, and horizontal axis is sample size as number of individuals). Parameters are raw, unconverted values directly from δaδi analyses.


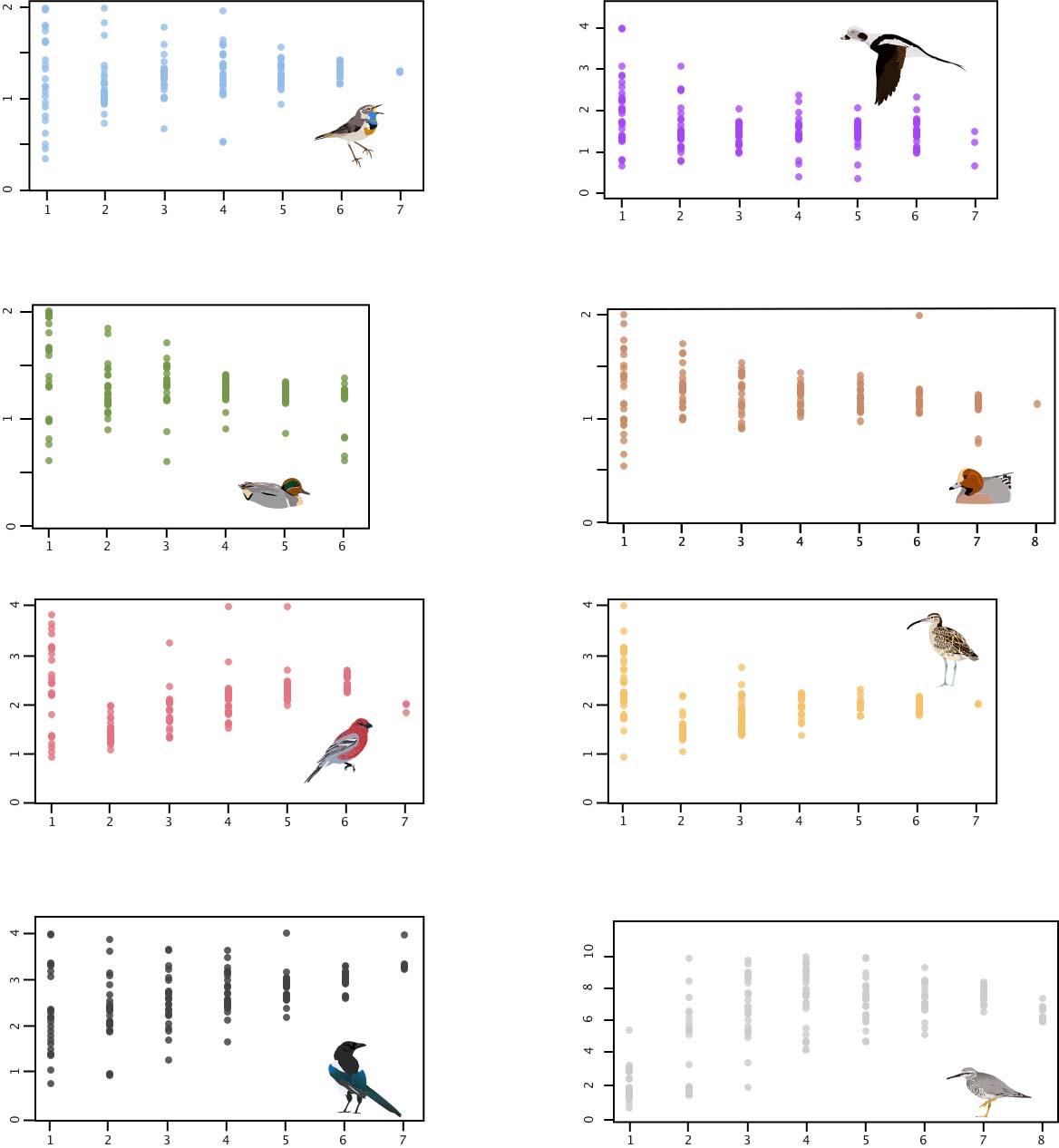


Figure S4: Estimates of *T* (time since divergence) at varying sample sizes in eight lineages (vertical axis is *T*, and horizontal axis is sample size as number of individuals). Parameters are raw, unconverted values directly from δaδi analyses.


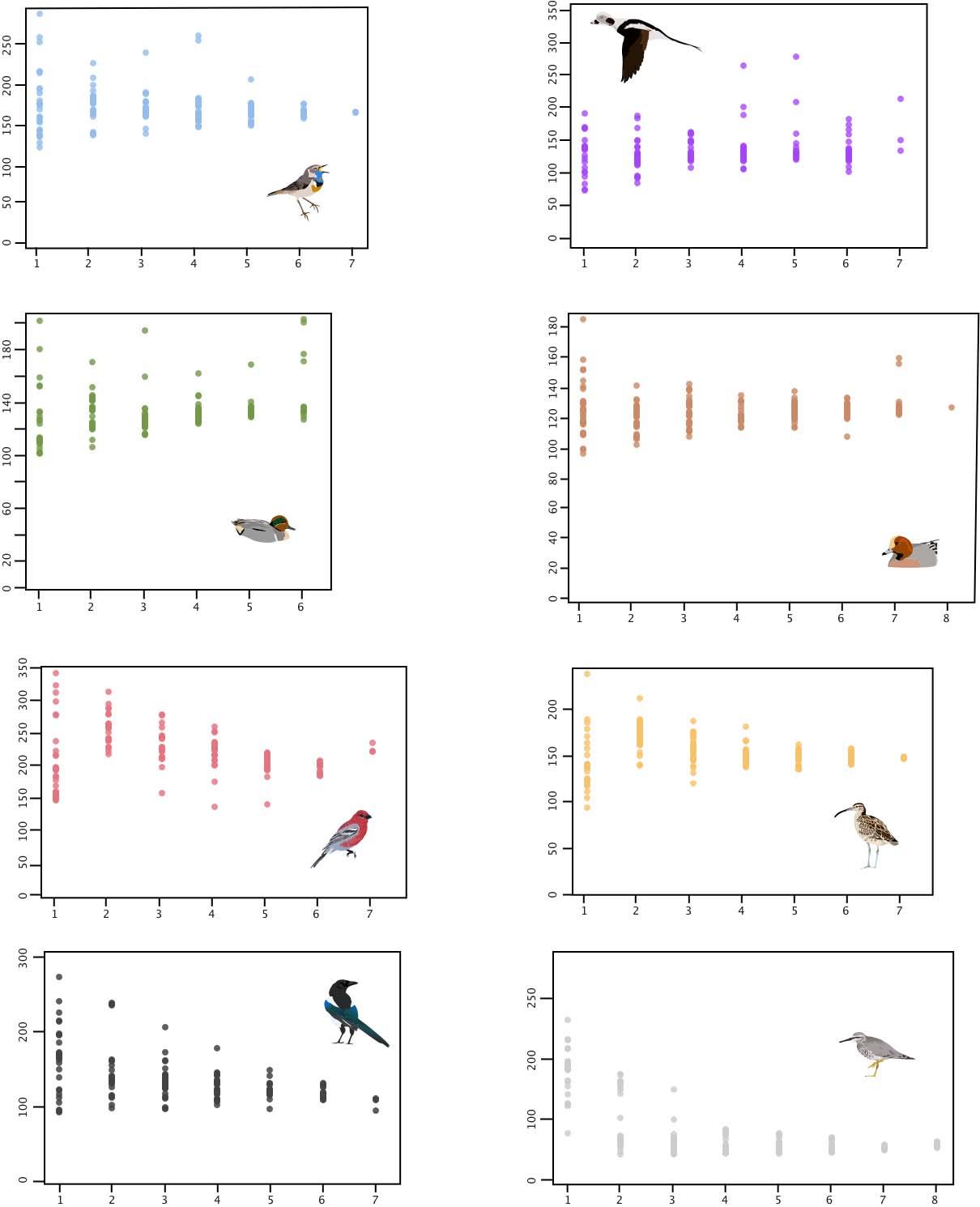


Figure S5: Estimates of *Θ* (4*N_ref_μ*) at varying sample sizes in eight lineages (vertical axis is *Θ*, and horizontal axis is sample size as number of individuals)**.** Parameters are raw, unconverted values directly from δaδi analyses.

**Figure S6.** Parameter-specific scaled root mean square error (SRMSE) values plotted against sample size (*N*). Note that vertical scales vary in each panel.

**Low-divergence lineages High-divergence lineages**

**
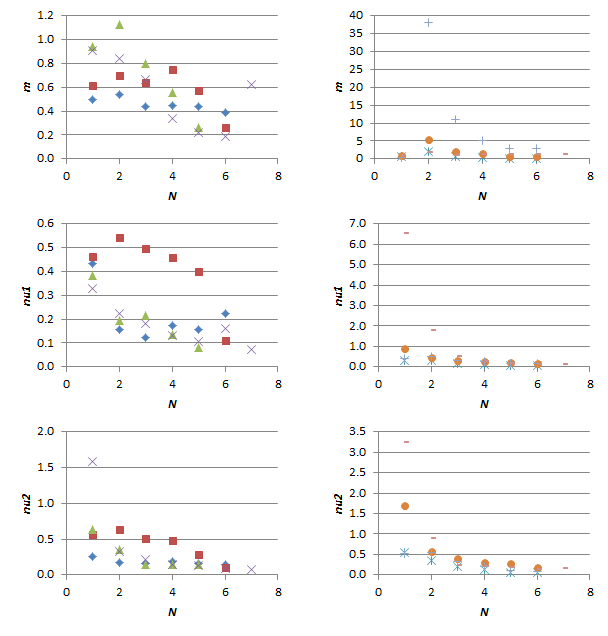
**

**Figure S6, continued.** Parameter-specific scaled root mean square error (SRMSE) values plotted against sample size (*N*). Note that vertical scales vary in each panel.

**Low-divergence lineages High-divergence lineages**

**
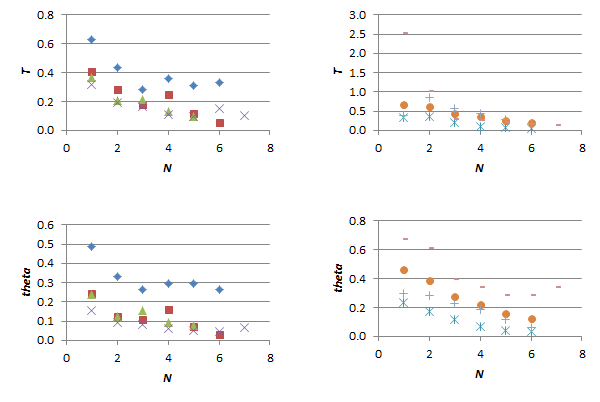
**
